# What Is the Functional Role of the Acyltransferase-like Domain in the Svx Peptidase of the Phytopathogenic Bacterium *Pectobacterium atrosepticum*?

**DOI:** 10.3390/ijms27094092

**Published:** 2026-05-02

**Authors:** Natalia Tendiuk, Roman Vasiliev, Anastasiya Diakonova, Olga Petrova, Olga Makshakova, Vladimir Gorshkov

**Affiliations:** 1 Kazan Institute of Biochemistry and Biophysics, FRC Kazan Scientific Center of RAS, 420111 Kazan, Russia; natalya.tendyuk@mail.ru (N.T.); vroma4713@gmail.com (R.V.);; 2 Institute of Fundamental Medicine and Biology, Kazan Federal University, 420008 Kazan, Russia

**Keywords:** plant infectious diseases, soft rot *Pectobacteriaceae*, *Pectobacterium atrosepticum*, extensin cleavage, extracellular proteases, substrate specificity, glycosylation pattern, Svx protein

## Abstract

The Svx protein is an established virulence factor in the phytopathogenic pectolytic bacterium *Pectobacterium atrosepticum* and is secreted into the host plant apoplast. However, its particular role has long remained enigmatic. In our recent studies, we showed that Svx proteins from pectolytic bacteria are metallopeptidases composed of two domains: peptidase and acyltransferase-like domains. Structural organization of the peptidase domain active site led us to hypothesize that its preferred substrates are extensins—hydroxyproline-rich glycoproteins of the plant cell wall. Nevertheless, direct experimental confirmation of extensin hydrolysis by Svx was lacking, and the precise role of the acyltransferase-like domain remained unclear. The present study aimed to address these issues. We showed that Svx indeed cleaves extensins while not degrading some other glycosylated and non-glycosylated proteins. The acyltransferase-like domain was shown to be critical for recognition of arabinan substituents in extensins, thereby providing optimal enzyme–substrate complementarity. Deletion of the acyltransferase-like domain abolished extensin hydrolysis by the truncated variant of Svx. Our study provides the first example of an apoplast-secreted protease from a phytopathogenic bacterium whose specificity toward specific target proteins (extensins) is achieved, at least in part, through structural elements that specifically recognize the distinctive glycosylation pattern of the target proteins.

## 1. Introduction

The plant cell wall serves as a central battleground in plant–pathogen interactions [1]. On one hand, it is intimately involved in most plant defense reactions, acting as both a physical barrier and a source of damage-associated molecular patterns (DAMPs) that trigger immunity [1,2,3,4]. On the other hand, cell wall components are primary targets for many (if not all) phytopathogenic microorganisms [5]. Pathogens degrade cell wall polymers to access nutrients sequestered within these structures and to disrupt tissue integrity, thereby facilitating effective spread within the host. Furthermore, because the cell wall functions as a sensor (monitoring integrity and detecting alterations via dedicated receptors), targeted manipulation of this compartment by pathogens can dampen defense responses and/or induce susceptible responses—reactions that promote pathogen colonization and symptom development [6,7,8]. However, well-documented examples in which pathogens deliberately degrade plant cell wall polymers specifically to suppress host immunity or induce susceptible responses (rather than solely for nutrient acquisition or mechanical penetration) remain relatively scarce in the literature, although such a strategy is biologically plausible. This strategy is most closely reflected not so much in the degradation of polysaccharides—the predominant components of the cell wall by mass—but rather in the hydrolysis of plant cell wall proteins.

Phytopathogens secrete extracellular proteases into the apoplast, which can degrade receptor-like proteins located in or associated with the plant cell wall, among other targets, thereby enabling the pathogen to evade detection by the host immune systems [9]. In addition, pathogen-secreted proteases can degrade structural proteins such as hydroxyproline-rich glycoproteins (HRGPs) extensins [10,11]. These proteins not only contribute to cell wall integrity through cross-linking but are also involved in signaling pathways that monitor cell wall functional state and coordinate physiological responses in accordance with that state [12]. Therefore, in addition to dampening plant cell wall integrity, cleavage of extensins by pathogen-secreted proteases can be presumed to lead to alterations in defense and susceptible responses, thereby promoting disease development. Additionally, since extensins are highly positively charged, it has been proposed that they may act as agglutinins toward negatively charged microbial cell surfaces [13]. Consequently, their degradation by pathogens could reduce this potential host-imposed negative effect on the microbe.

Virtually no detailed information is available on the substrate specificity of extracellular proteases from phytopathogenic bacteria toward specific plant cell wall proteins, and it is generally assumed that these enzymes act non-specifically on cell wall proteins, despite the substantial differences in their structures, including glycosylation patterns [14,15]. The issue of substrate specificity of bacterial extracellular proteases was raised (but not conclusively proven) in our previous studies characterizing the Svx proteases of pectolytic bacteria [16,17].

Pectolytic bacteria belonging to the soft rot *Pectobacteriaceae* are considered among the most harmful phytopathogens [18,19]. Their major virulence factors are plant cell wall-degrading enzymes (PCWDEs), primarily pectinases, which destroy the middle lamella, resulting in host plant tissue maceration [20]. However, the action of pectinases alone is insufficient to ensure disease development, and additional virulence factors are required for full virulence of these bacteria [8,21]. Among these additional virulence factors are Svx proteins. In *Pectobacterium atrosepticum*, the Svx protein has been shown to be required for full virulence, to be secreted via the type II secretion system into the apoplast, and its production to be activated by quorum sensing and in the presence of plant metabolites [22,23]. Prior to our earlier studies, however, no information existed on the specific functions of Svx proteins. In our previous work, we demonstrated that Svx proteins of pectolytic bacteria (*P. atrosepticum* and *Dickeya solani*) are gluzincin metallopeptidases with a conserved tertiary structure comprising two domains: a peptidase domain and an acyltransferase-like domain [16,17]. The peptidase domain has an in silico-predicted structure that enables its interaction with α-glycosylated proteins, which, among the diverse proteins of the plant cell wall, are represented by extensins. The physiological effects of Svx proteins are manifested in their ability to induce ethylene-regulated responses in the host plant, which play a dominant role in susceptibility to *P. atrosepticum* [24]. Nevertheless, the Svx-mediated extensin hydrolysis had not been demonstrated experimentally, nor had the acyltransferase-like domain’s function been clarified. Therefore, the aim of the present study was to elucidate the functional role of the acyltransferase-like domain of the *P. atrosepticum* Svx protein and to experimentally test its capacity to hydrolyze extensins.

## 2. Results

### 2.1. Docking Simulations of Extensin Arabinan Side Chains with Svx Protein of P. atrosepticum (Pba Svx)

Since extensin glycosylation encompasses not only α-galactosylation of serine residues but also the attachment of short oligoarabinan chains (one to four arabinofuranose residues) to clusters of consecutive hydroxyproline residues [25], we hypothesized that the acyltransferase-like domain of *Pba* Svx, in addition to contributing to the formation of the negatively charged pocket surrounding the peptidase domain active site [17], mediates specific interactions with these arabinan chains, thereby facilitating proper substrate orientation and positioning within the active site for efficient hydrolysis. To test this hypothesis, we performed molecular docking of *Pba* Svx with a set of conformers representing the longest characteristic extensin arabinan chain, namely the tetraarabinofuranoside (α-L-Araf(1→3)-β-L-Araf(1→2)-β-L-Araf(1→2)-β-L-Araf) using AutoDock 4.2 software.

**Figure 1:**
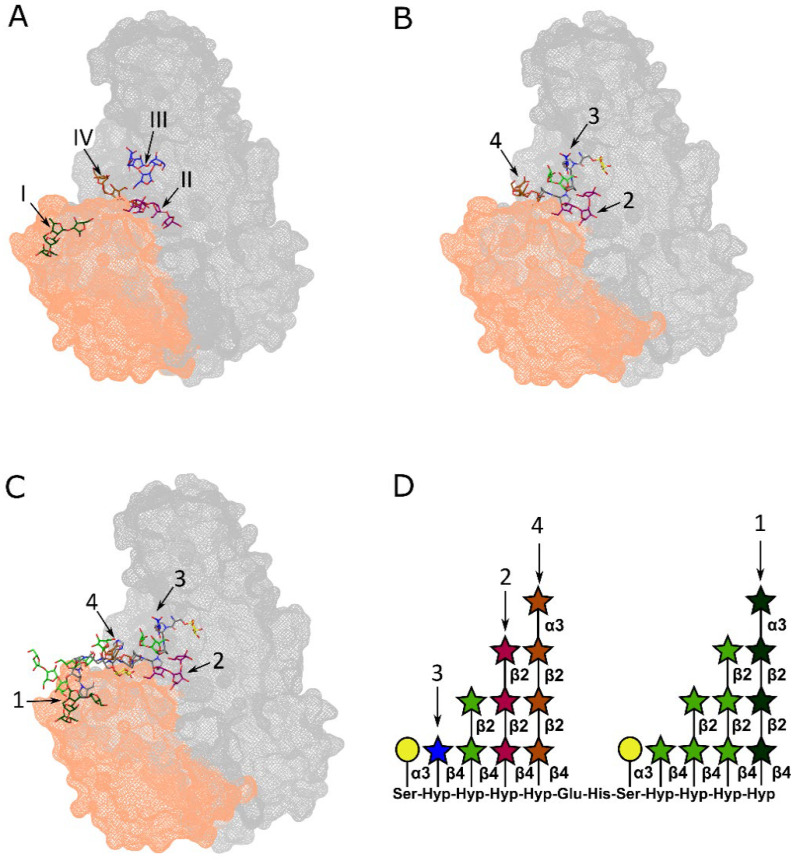
Modeling of interactions between arabinans and the Svx protein of *Pectobacterium atrosepticum* (*Pba* Svx). (**A**) Superpositions of four representative docking poses for the arabinan chain (α-L-Araf1-3β-L-Araf1-2β-L-Araf1-2β-L-Araf) within the four most energetically favorable arabinan-binding sites on the surface of *Pba* Svx (binding energies: I—−3.05 kcal/mol; II—−1.45 kcal/mol; III—−1.28 kcal/mol; IV—−0.84 kcal/mol). (**B**) Superposition of an extensin characteristic glycosylated fragment consisting of five amino acid residues (SPPPP); galactose residue is shown in yellow, arabinan chains shown in light green do not interact with the surface of *Pba* Svx. (**C**) Superposition of a glycosylated fragment of the carrot extensin (Uniprot ID P06599) consisting of twelve amino acid residues (38-SPPPPEHSPPPP); galactose residues are shown in yellow, arabinan chains shown in light green do not interact with the surface of *Pba* Svx. (**D**) A scheme of the glycosylated peptide ligand (38-SPPPPEHSPPPP) (Uniprot ID P06599), ref. [25] used for the modeling of *Pba* Svx-extensin interactions; the yellow circle indicates a galactose residue; stars represent arabinose residues; the numbered arabinose chains correspond to the same numbering designated in the *Pba* Svx-extensin interaction models (panels (**B**,**C**)). Peptidase and acyltrasferase-like domains of the Svx protein are shown in gray and orange color, respectively. O atoms are given in red, N—in blue, and C atoms of peptide chain—gray.

Clustering of the 200 docked poses of the arabinan chains (within a 10 Å RMSD cutoff) revealed four preferred binding epitopes on the surface of *Pba* Svx. These epitopes were distributed across both the peptidase domain and the acyltransferase-like domain, suggesting potential roles for both domains in arabinan recognition and binding. The most favorable arabinan binding site (binding energy −3.05 kcal/mol) was observed on the acyltransferase-like domain surface (arabinan chain is shown in dark green, Figure 1A), involving residues Val569, Lys570, Ser554, Ala555, Ser556, Glu566, Gln557, and Phe560. The second most favorable epitope (−1.45 kcal/mol) was revealed at the boundary between the peptidase and acyltransferase-like domains (arabinan chain is shown in magenta, Figure 1A), involving residues Arg551 Gly552, and Phe563. The other two binding sites (blue and ochre arabinan chains, Figure 1A) were located on the peptidase domain and formed by the residue sets Phe526, Met527, Tyr166, Met162, Tyr 532 and Trp413, Asp414, Arg177, Tyr178, respectively (binding energies −1.28 and −0.84 kcal/mol, respectively).

The docked arabinan chains were oriented toward each other with their reducing ends facing in close proximity. This arrangement allows us to propose that, when covalently attached to extensin glycoproteins, multiple arabinan side chains on contiguous hydroxyproline residues might serve as spatial guides or anchors, thereby enabling the proper positioning of O-galactosylated serine residues within the catalytic site of the peptidase domain and facilitating efficient proteolytic cleavage.

To test this hypothesis, we first positioned a glycosylated extensin fragment, corresponding to repeating motif (SPPPP) (where P stands for hydroxyproline) characteristic of all extensins onto the *Pba* Svx structure such that arabinan side chains were superimposed onto the docked arabinan conformers, with the Ser-O-galactose moiety positioned within the active site of the peptidase domain, following our previous structural predictions [16] (Figure 1B). At this positioning, the arabinans attached to the hydroxyproline residues interacted with three of the four most favorable arabinan-binding sites on *Pba* Svx, while the most energetically favorable arabinan-binding site remained distant and not included into interactions with the glycopeptide fragment (Figure 1B). This indicates that the presence of arabinan chains not only does not hinder extensin interaction with the active site of the *Pba* Svx peptidase domain but may actually facilitate it through interactions with the arabinan chains, despite the fact that arabinan-binding site with the highest affinity does not participate in this interaction.

Then, we positioned a longer extensin fragment containing two arabinan-substituted clusters of contiguous hydroxyproline residues (38-SPPPPEHSPPPP from well-characterized carrot extensin (UniProt ID P06599)). In this case, all four of the most favorable arabinan-binding sites of *Pba* Svx engaged with the arabinan-substituted extensin oligopeptides, with no steric clashes observed between the arabinan chains and the enzyme (Figure 1C). These results suggest that the two sequential arabinan-substituted clusters of contiguous hydroxyproline residues (Figure 1D) provide the optimal enzyme–substrate interaction, promoting the correct positioning of O-galactosylated serine residues within the catalytic site of the peptidase domain.

### 2.2. Svx Peptidase Degrades Plant Extensins

**Figure 2:**
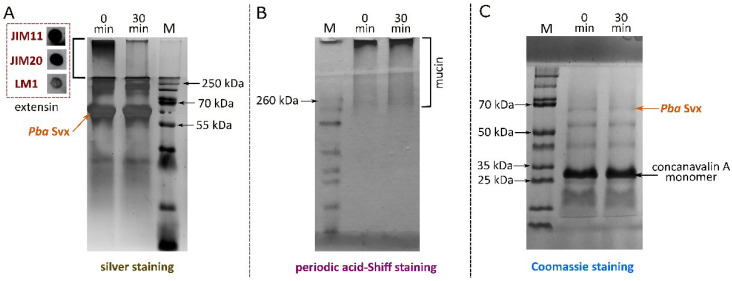
SDS-PAGE electrophoregrams of the extensin-enriched fraction (**A**), porcine gastric mucin (**B**) and concanavalin A (**C**) after 0 and 30 min of incubation with the *Pectobacterium atrosepticum* Svx protein (*Pba* Svx). In panel (**A**), the region delimited by the dashed box shows immunolabeling of the extensin-enriched fraction with the JIM11, JIM20 and LM1 antibodies. The most appropriate staining method was used for each substrate: silver staining (**A**), periodic acid–Schiff (PAS) staining (**B**) and Coomassie blue staining (**C**). M, molecular weight marker.

To experimentally test the predicted ability of *Pba* Svx to hydrolyze extensins, an extensin-enriched fraction was isolated from carrot (*Daucus carota*) roots. On SDS-PAGE, the preparation appeared as a dominant high-molecular-mass band that failed to enter the gel, consistent with the heavily glycosylated and cross-linked nature of mature extensins (Figure S1). The protein-to-carbohydrate ratio in the obtained extensin-enriched fraction was approximately 1:1 (*w*/*w*). Monosaccharide analysis revealed that arabinose and galactose comprised 82% and 12% of the total carbohydrate mass, respectively. These compositional features are consistent with those of extensin preparations described previously [26]. Immunoblotting with three monoclonal antibodies specific for extensin epitopes (JIM11, JIM20, and LM1) confirmed the presence of typical extensin glyco-epitopes in the obtained preparation (Figure 2).

Incubation of the extensin-enriched fraction with recombinant *Pba* Svx (30 min, 40 °C) resulted in pronounced degradation of the high-molecular-mass extensin band, evidenced by a dramatic reduction in staining intensity at the top of the gel (Figure 2). In contrast, under identical conditions, *Pba* Svx did not degrade the O-glycosylated porcine gastric mucin (which carries α-GalNAc-Ser/Thr rather than α-Gal-Ser and lacks arabinan chains) or the non-glycosylated plant lectin concanavalin A, which possesses a regular structure.

### 2.3. The Acyltransferase-like Domain Is Essential for Extensin Hydrolysis but Dispensable for the General Peptidase Activity of Svx Peptidase

**Figure 3:**
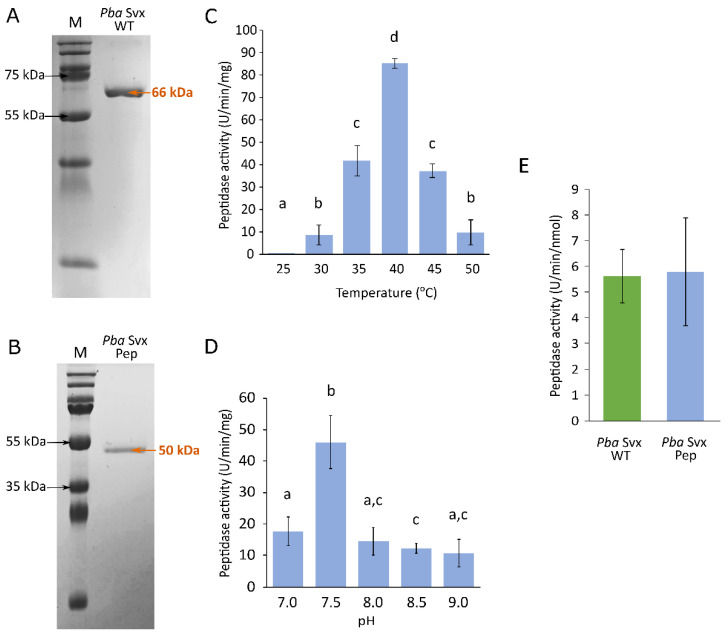
The characteristics of the truncated variant of the *Pectobacterium atrosepticum* Svx protein lacking the acyltransferase-like domain and consisting essentially of the catalytic peptidase domain (*Pba* Svx Pep). (**A**,**B**) SDS-PAGE electrophoregrams of the purified recombinant full-length wild-type protein (*Pba* Svx WT) and the truncated mutant protein (*Pba* Svx Pep), respectively. (**C**,**D**) The peptidase activity of *Pba* Svx Pep toward azocasein at different pH values (determined at 37 °C) and different temperatures (determined at pH 7.5), respectively. Significant differences between the activities measured at different temperatures or pH values were determined using the Mann–Whitney test with Benjamini–Hochberg adjustment, FDR < 0.05. Mean values denoted by different letters are significantly different from each other. (**E**) Comparison of peptidase activities of *Pba* Svx WT and *Pba* Svx Pep measured under optimal conditions for both proteins (40 °C, pH 7.5). Activities were analyzed in six replicates. No significant difference was found between the peptidase activities of *Pba* Svx WT and *Pba* Svx Pep (Mann–Whitney two-sided U test, *p* > 0.05).

To directly assess the functional contribution of the acyltransferase-like domain to the extensin-hydrolyzing activity of *Pba* Svx, a truncated variant lacking this domain (*Pba* Svx Pep, comprising essentially only the catalytic peptidase domain) was obtained. *Pba* Svx Pep retained peptidase activity toward the flexible substrate azocasein. The most favorable conditions for azocasein hydrolysis were pH 7.5 and 45 °C (Figure 3). These optima were similar to those previously determined for the wild-type *Pba* Svx protein [16]. The peptidase activity against azocasein did not differ between *Pba* Svx and *Pba* Svx Pep (Figure 3).

**Figure 4:**
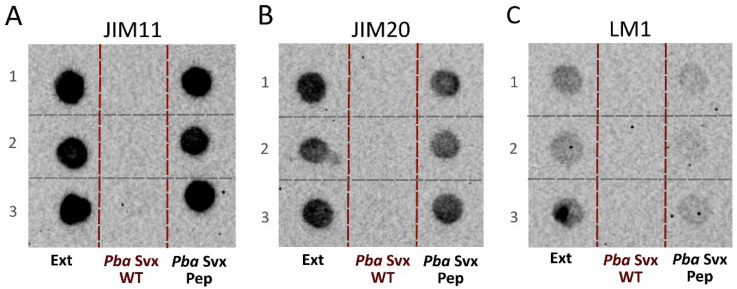
Immunolabeling of the extensin-enriched fraction after 30 min of incubation alone (Ext) or with wild-type full-length *Pectobacterium atrosepticum* Svx protein (*Pba* Svx WT) or the truncated variant lacking the acyltransferase-like domain and consisting essentially of the catalytic peptidase domain (*Pba* Svx Pep). Incubation was performed at 40 °C, pH 7.5. Immunolabeling was carried out using the extensin-specific antibodies JIM11 (**A**), JIM20 (**B**) and LM1 (**C**). Lanes 1, 2 and 3 represent three analytical replicates.

However, in contrast to the wild-type *Pba* Svx, the mutant protein *Pba* Svx Pep lost the ability to degrade extensins (Figure 4). After 30 min of incubation with *Pba* Svx, immunolabelling of extensins with JIM11, JIM20 and LM1 antibodies was almost completely abolished, whereas the signal intensity in samples incubated with *Pba* Svx Pep remained comparable to that of the control (extensin preparation incubated alone for 30 min) (Figure 4).

### 2.4. The Truncated Variant of Svx Peptidase Lacking the Acyltransferase-like Domain Fails to Induce Host Ethylene-Regulated Susceptible Responses

**Figure 5:**
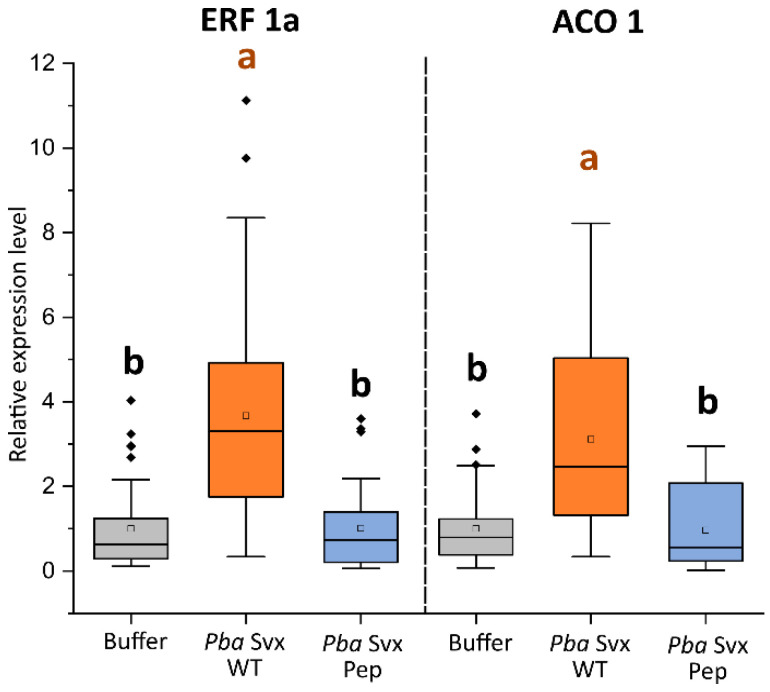
The expression levels of genes encoding the ethylene-regulated transcription factor ERF1a and 1-aminocyclopropane oxidase (ACO1) in tobacco leaves following mock infiltration (buffer, gray box plots) or infiltration with wild-type full-length *Pectobacterium atrosepticum* Svx protein (*Pba* Svx WT, orange box plots) or the truncated variant lacking the acyltransferase-like domain and consisting essentially of the catalytic peptidase domain (*Pba* Svx Pep, blue box plots). The expression level of both genes in mock-infiltrated plants was set to 1. Expression was analyzed in at least twenty biological replicates. Statistical significance was determined by the Kruskal–Wallis test followed by Dunn’s post-test (*p* < 0.05). Different letters above the bars indicate statistically significant differences between the experimental groups.

Since *Pba* Svx has been previously shown to induce plant susceptible responses, specifically ethylene-regulated reactions whose activation is crucial for *P. atrosepticum*-caused disease development [17], we tested whether the truncated variant of *Pba* Svx lacking the acyltransferase-like domain (*Pba* Svx Pep) is able to induce these reactions following infiltration into tobacco leaves. Infiltration of wild-type *Pba* Svx into tobacco leaves resulted in the upregulation of genes encoding the ethylene-regulated transcription factor ERF1 and 1-aminocyclopropane oxidase (an ethylene biosynthetic enzyme, ACO) by 3.5- and 3.0-fold, respectively. However, no such upregulation was observed following infiltration of the mutant *Pba* Svx Pep (Figure 5).

## 3. Discussion

The present study aimed to elucidate the functional role of the acyltransferase-like domain in the Svx peptidase of *Pectobacterium atrosepticum* (*Pba* Svx) and to provide experimental evidence for its previously predicted ability to hydrolyze plant extensins. The Svx protein is an established virulence factor in *P. atrosepticum*: mutants deficient in *svx* gene exhibit reduced virulence [22]. It has been demonstrated that Svx is secreted via the type II secretion system [22] and is thus delivered from the bacterium into the plant apoplast during host colonization, where its activity is manifested. In our recent work, we showed that Svx proteins from pectolytic bacteria (*P. atrosepticum* and *Dickeya solani*) are gluzincin metallopeptidases characterized by a conserved tertiary structure comprising two domains: a catalytic peptidase domain and an additional acyltransferase-like domain [16,17]. Structural analysis of the peptidase domain’s active site led us to hypothesize that its preferred substrates are extensins—hydroxyproline-rich glycoproteins (HRGPs) of the plant cell wall (PCW) that contribute to structural integrity through cross-linking and also participate in signaling pathways due to their association with regulatory kinases [12].

Prior to the current study, however, direct experimental confirmation of extensin hydrolysis by Svx was lacking, and the precise role of the acyltransferase-like domain remained unclear. The only available information was that this domain, together with the peptidase domain, contributes to the formation of a negatively charged pocket around the catalytic site, which could potentially facilitate interaction with positively charged extensins [17]. Our earlier prediction of *Pba* Svx substrate specificity relied primarily on the finding that the active site of the peptidase domain is structurally arranged to facilitate interaction with α-galactosylated serine residues, which represent a characteristic feature of extensin glycosylation motif [16]. However, extensin glycosylation also includes short oligoarabinan chains (typically one to four arabinofuranose residues) attached to clusters of contiguous hydroxyproline residues [25]. We therefore hypothesized that the acyltransferase-like domain plays a key role in recognizing these arabinan chains, thereby significantly contributing to the overall substrate specificity of *Pba* Svx.

Molecular docking simulations supported this idea. Four preferred binding epitopes for tetraarabinofuranoside chains were identified on the *Pba* Svx surface, distributed across both domains. The most energetically favorable site was located on the acyltransferase-like domain, while two others mapped to interfacial regions formed jointly by the peptidase and acyltransferase-like domains and one epitope mapped to the surface of peptidase domain. Notably, in these docking poses the bound arabinan chains were oriented toward each other with their reducing ends in close proximity. This arrangement suggested that, in the context of native extensins, multiple arabinan side chains could simultaneously engage the enzyme surface rather than a single arabinan chain.

To model a more biologically relevant interaction, we positioned glycosylated extensin fragments from carrot extensin onto the *Pba* Svx structure. When a short fragment (SPPPP) containing an α-galactosylated serine followed by four hydroxyproline residues bearing arabinan chains was used, the arabinans interacted with three of the four identified binding sites and did not sterically hinder placement of the O-galactosylated serine in the peptidase active site; instead, they appeared to stabilize the correct orientation. However, the highest-affinity arabinan-binding site (on the acyltransferase-like domain) did not participate in this interaction. In turn, when a longer fragment containing two sequential clusters of arabinan-substituted hydroxyproline repeats (SPPPPEHSPPPP) was modeled, all four binding sites—including the most favorable one on the acyltransferase-like domain—engaged with the arabinan chains without steric clashes. These results suggest that the acyltransferase-like domain participates in the recognition of arabinan substituents in extensins and that two sequential arabinan-substituted clusters of contiguous hydroxyproline residues provide optimal enzyme–substrate complementarity, promoting precise positioning of O-galactosylated serine residues within the peptidase catalytic site.

To experimentally validate the in silico-predicted role of the acyltransferase-like domain in extensin hydrolysis, we obtained (1) recombinant full-length *Pba* Svx; (2) a truncated mutant lacking the acyltransferase-like domain (*Pba* Svx Pep, consisting essentially of the peptidase domain only); and (3) an extensin-enriched protein fraction from carrot roots. Removal of the acyltransferase-like domain did not impair the enzyme’s ability to hydrolyze the general protease substrate azocasein; peptidase activity of *Pba* Svx Pep was comparable to that of wild-type *Pba* Svx. However, unlike full-length *Pba* Svx, which degraded extensins, *Pba* Svx Pep failed to hydrolyze extensins despite retaining proteolytic activity toward the flexible substrate (azocasein). This demonstrates that *Pba* Svx is capable of cleaving extensins and that the acyltransferase-like domain is essential for this activity. However, although the acyltransferase-like domain is required for extensin degradation, it is dispensable for the hydrolysis of an intrinsically disordered protein lacking a defined tertiary structure, such as azocasein. These results suggest that the hydrolysis of extensins, which possess a rigid, extended rod-like conformation, requires stabilization and precise positioning of the substrate within the Svx active site. This stabilization is provided, at least in part, by the interaction of the acyltransferase-like domain with the arabinan substituents of extensins. In turn, the absence of a defined tertiary structure allows the substrate to be properly positioned within the catalytic domain of Svx without the need for additional stabilization provided by structural elements of either the enzyme (acyltransferase-like domain) or the substrate (arabinan chains).

The inability of *Pba* Svx Pep to degrade extensins was associated with its failure to induce ethylene-regulated responses upon infiltration into plant leaves—reactions that represent a susceptible response during *P. atrosepticum* infection [24] and are triggered by full-length Svx [17]. Thus, loss of the acyltransferase-like domain by *Pba* Svx abolishes not only extensin hydrolysis ability but also the downstream physiological effect that promotes host susceptibility. It is likely that Svx-mediated induction of ethylene-regulated responses is linked to the signaling function of extensins. This signaling function is largely determined by the functional interplay of extensins with regulatory kinases that coordinate plant responses [12]. Consequently, the inability of the truncated variant *Pba* Svx Pep to degrade extensins (due to the lack of the acyltransferase-like domain) suppresses its downstream physiological effect on the host plant. A more complete characterization of Svx-mediated plant responses, together with the identification of all components involved in the signal transduction pathway represents an intriguing direction for future research on the mechanism of action of this virulence factor.

To assess substrate specificity, we tested two other proteins in addition to extensins: non-glycosylated plant lectin with regular tertiary structure, concanavalin A (ConA), and porcine gastric mucin (a heavily O-glycosylated mammalian glycoprotein). Porcine mucin was chosen as a control protein because a structurally similar bovine mucin is a known substrate of the glycopeptidase from *Clostridium perfringens*, whose active site structure most closely resembles that of *Pba* Svx. It was this structural similarity in the active sites that led us, in our previous study, to hypothesize that *Pba* Svx might act as a glycopeptidase [16]. Similar to extensins, mucin has α-substituted serine residues; however, in extensins the substituent is galactose, while in mucin it is N-acetylgalactosamine, which is further extended by two or more additional sugar residues [25,27]. In addition, mucin does not contain arabinan chains. Despite the similarity between the peptidase active sites of the *C. perfringens* glycopeptidase and *Pba* Svx, the two enzymes differ substantially overall: the *C. perfringens* enzyme lacks the acyltransferase-like domain and instead has four carbohydrate-binding domains (two CBM32 and two CBM51) [28], indicating that their substrate specificity can differ.

*Pba* Svx failed to degrade either ConA or porcine mucin under the same conditions where it completely hydrolyzed the carrot extensin preparation. This indicates that *Pba* Svx does not simply degrade extensins alongside other proteins, but is specifically targeted toward extensins. Most probably, this specificity is determined, at least in part, by the distinctive glycosylation pattern of extensins—namely, the presence of short oligoarabinan chains attached to clusters of consecutive hydroxyproline residues. In contrast, concanavalin A is a non-glycosylated protein, whereas porcine mucin carries a different type of heavy O-glycosylation based on GalNAc and lacks arabinan chains. Although we cannot exclude the contribution of amino acid sequence or other structural features unrelated to glycosylation, the complete absence of activity of *Pba* Svx toward ConA and porcine mucin, combined with the functional importance of the acyltransferase-like domain for the interaction with arabinan chains, supports the idea that recognition of the arabinan substituents plays a key role in *Pba* Svx substrate specificity. α-Galactosylation of serine residues, another characteristic feature of extensin glycosylation, may also contribute to this specificity [16,17], although this contribution appears to be independent of the acyltransferase-like domain.

Two extracellular proteases secreted by phytopathogenic bacteria into the plant apoplast have previously been shown to degrade extensins: the Prt1 protease from *Pectobacterium carotovorum* and the gp120-degrading protease from *Xanthomonas campestris* pv. *campestris* [10,11]. However, unlike *Pba* Svx, Prt1 acts in a relatively non-specific manner and cleaves a large number of proteins, including extensins. Its substrate preference is dictated primarily by the amino acid sequence, with cleavage occurring between a proline residue and a hydrophobic amino acid (Ala, Val or Phe)—a motif that is widespread in many plant proteins [11]. By contrast, the gp120-degrading protease exhibits narrow substrate specificity, degrading only a limited set of hydroxyproline-rich glycoproteins, including extensins [10]. Nevertheless, the molecular mechanisms underlying this specificity, particularly those related to the structural organization of gp120-degrading protease itself, remain unknown. To the best of our knowledge, our study provides the first example of an apoplast-secreted protease from a phytopathogenic bacterium whose specificity toward specific target proteins (extensins) is achieved, at least in part, through structural elements that specifically recognize the distinctive glycosylation pattern of the target proteins.

## 4. Materials and Methods

### 4.1. In Silico Analysis of Arabinan Binding to the Svx Protein

A set of conformers of the extensin arabinan chain (α-L-Araf(1→3)-β-L-Araf(1→2)-β-L-Araf(1→2)-β-L-Araf) was generated from a molecular dynamics trajectory calculated using the Glycam06j force field [29] and the AMBER 2025 program [30]. The tetrasaccharide was immersed in a periodic boundary box containing TIP3P water molecules. The simulations were performed in the isothermal–isobaric thermodynamic ensemble at 300 K. Temperature and pressure were maintained constant using a Langevin thermostat (collision frequency 2 ps^−1^) and a weak coupling algorithm (relaxation time 2 ps), respectively. An integration time step of 2 fs was employed, along with the SHAKE algorithm [31] to constrain bonds involving hydrogen atoms. For long-range electrostatic interactions, the Particle Mesh Ewald (PME) method was applied [32]. The system was initially minimized over 5000 steps, followed by a phase of equilibration. During the production phase, a total of 200 ns of trajectory data were collected. Ten conformers, evenly distributed throughout the trajectory, were selected for subsequent docking analyses. Docking of α-L-Araf(1→3)-β-L-Araf(1→2)-β-L-Araf(1→2)-β-L-Araf to *Pba* Svx was performed using AutoDock 4.2 [33]. The structure of *Pba* Svx was obtained as described previously [17]. The grid was centered on the active site region and the extended β-structure-rich acetyltransferase-like domain. Among all poses, the top two hundred positions were clustered using a 10 Å RMSD cut-off. The representative conformations of the most populated and energetically favorable clusters were considered the most likely binding modes.

The glycosylated fragments (SPPPP and 38-SPPPPEHSPPPP) of carrot extensin (Uniprot ID P06599) were positioned in the active site of *Pba* Svx so that the arabinan chains overlapped with those obtained from docking, and the Ser-O-Gal residue was placed in the active site according to our previous structural estimations [16]. The resulting complex was energy minimized in Maestro (Schrödinger Release 2016-1: Maestro, Schrödinger, LLC, New York, NY, USA, 2016).

### 4.2. Production of Recombinant Proteins

The purified preparation of the wild-type Svx protein of *Pectobacterium atrosepticum* SCRI1043 (WP_011092533.1) (*Pba* Svx) was obtained according to the protocol published previously [16]. The purified preparation of the truncated mutant *Pba* Svx protein (*Pba* Svx Pep) deficient in the acyltransferase-like domain and containing only the peptidase domain was obtained as follows. The part of the *svx* gene (ECA0931) corresponding to the peptidase domain coding sequence was PCR-amplified from the previously obtained recombinant vector pET-51bΔ*svx* [16] using primers SvxPep_F: CAGCTACCATGGCCGCTGAAGCTTGCG and SvxPep_R: GTCATGGAGCTCCTACTTTTCGAACTGCGGGTGGCTCCATCCTTCAGGCCAAACGCCAGT. The obtained PCR product with C-terminal Strep-tag II was ligated into the pET-51b vector (Merck KGaA, Darmstadt, Germany).

Cells of *Escherichia coli* strain BL21 (DE3) (Invitrogen, Waltham, MA, USA) were transformed with the resultant pET51b-SvxPep plasmid and grown at 37 °C in 2 L of sterile LB:M9 (1:1) media supplemented with 100 µg/mL ampicillin until the culture reached a density of ~0.6 at 600 nm. Recombinant protein synthesis was induced by the addition of isopropyl-β-D-1-thiogalactopyranoside (IPTG) (Thermo Fisher Scientific, Waltham, MA, USA) to a final concentration of 1 mM. Cultures were kept for 3 h at 37 °C with shaking. Bacterial cells were collected, and cell lysis was performed using BugBuster Protein Extraction Reagent (Novagen, Temecula, CA, USA), 0.1 mg/mL of lysozyme (Thermo Fisher Scientific, Waltham, MA, USA), and 0.1 mg/mL of DNaseI (diaGene, Moscow, Russia) at 37 °C for 20 min. Cell lysates were centrifuged (12,000× *g*, 4 °C, 30 min), and the insoluble fraction (inclusion bodies) was washed with the buffer (100 mM Tris-HCl, pH 8.0, 150 mM NaCl) and pelleted again (12,000× *g*, 4 °C, 30 min). The pellet was dissolved in a denaturing buffer (100 mM Tris-HCl, 150 mM NaCl, 8 M urea). Then, one volume of the resuspended inclusion bodies was added dropwise to 20 volumes of the refolding buffer (100 mM Tris-HCl, 150 mM NaCl, 1.25 mM reduced glutathione, 1.25 mM oxidized glutathione, 1 M arginine, and 1 mM ZnSO_4_) at 4 °C for 12 h. The resultant solution was clarified by centrifugation at 10,000× *g* for 30 min. The refolded protein was loaded in parts on the chromatography column with Strep-Tactin XT 4Flow High Capacity resin (IBA-Lifesciences, Göttingen, Germany). The one-step purification was carried out at 4 °C. The mutant form *Pba* Svx Pep was eluted with the buffer (100 mM Tris-HCl, 150 mM NaCl, 50 mM biotin) and used in experiments.

### 4.3. Monosaccharide Analysis

Monosaccharide analysis was performed using high-performance anion exchange chromatography on an ICS6000 system (Thermo Fisher Scientific, Waltham, MA, USA) and pulse-amperometric detection (Waveform A). To break the glycosidic bonds, the samples were hydrolyzed with 2 M trifluoroacetic acid at 120 °C for 1 h. The resulting monosaccharides were separated on a CarboPac PA-1 column (4 × 250 mm, Thermo Fisher Scientific, Waltham, MA, USA). Neutral monosaccharides were separated isocratically for 20 min using 0.015 M NaOH. To separate uronic acids, a linear gradient was used according to the following scheme: 20–21 min—until the ratio of eluents A:B = 90%:10%; 21–31 min—until the ratio of eluents A:B = 70%:30%, where eluent A is 0.015 M NaOH, and eluent B is 1 M NaOAc in 0.1 M NaOH. The column temperature was 30 °C, the flow rate was 1 mL/min. Before each subsequent analysis, the column was washed and equilibrated in the mode 100% B for 10 min, 100% A for 30 min. Quantitative determination of monosaccharides was carried out using calibration curves for commercial monosaccharides (Merck KGaA, Darmstadt, Germany), pre-treated similarly to the analyzed samples. Data analysis was performed using Chromeleon 7.0 software (Thermo Fisher Scientific, Waltham, MA, USA).

### 4.4. Extraction of the Extensin-Enriched Fraction

The extensin-enriched fraction was obtained according to the published protocol [34]. Twenty grams of carrot (*Daucus carota*) disks were incubated for 72 h at 30 °C and 180 rpm in 2 L flasks containing 200 mL of 5 mM potassium phosphate buffer, pH 6.0, supplemented with 50 µg/mL chloramphenicol. The disks were washed with deionized water daily, with a change of the incubation medium. The disks were homogenized in liquid nitrogen using a mortar and pestle. The extraction and purification of extensins were performed at 4 °C. The obtained homogenate was shaken for 1 h in 30 mL of ice-cold lysis buffer (50 mM sodium acetate, pH 4.6, 0.7 M sucrose, 1% DTT, 1 mM PMSF), and the suspension was then filtered through a Büchner funnel with filter paper into a Bunsen flask. The pellet was then washed twice with 500 mL of 50 mM sodium acetate (pH 4.6) buffer and incubated on ice in 200 mL of CaCl_2_-extraction buffer (50 mM sodium acetate, pH 4.6, 0.2 M CaCl_2_, 1 mM PMSF) on a shaker overnight. The suspension was filtered and the CaCl_2_-extracted fraction was collected. The remaining pellet was incubated on ice in 200 mL of LiCl extraction buffer (50 mM sodium acetate, pH 4.6, 3 M LiCl, 1 mM PMSF) on a shaker overnight. The suspension was filtered and the LiCl-extracted fraction was collected. In both the CaCl_2_-extracted and LiCl-extracted fractions, non-glycosylated proteins were precipitated with 10% trichloroacetic acid (TCA). After precipitation, the supernatants were concentrated using an Amicon Ultra Centrifugal Filter with a 10 kDa MWCO (Merck KGaA, Darmstadt, Germany) to 2 mL, dialyzed against 100 mL of 20 mM Tris-HCl (pH 8.0), and then the processed CaCl_2_-extracted and LiCl-extracted fractions were combined. The obtained 4 mL of mixed fraction was applied to a 1 mL column of CM-Sepharose Fast Flow (Sigma-Aldrich, St. Louis, MO, USA) equilibrated with 20 mM Tris-HCl (pH 8.0). The column was washed with 2 column volumes (CV) of 20 mM Tris-Cl (pH 8.0). The extensin-enriched fraction was eluted with elution buffer (20 mM Tris-Cl, pH 8.0, 1 M NaCl). All protein fractions obtained during purification were visualized by SDS-PAGE [35] followed by silver staining, because highly glycosylated proteins cannot be stained properly by Coomassie Brilliant Blue [36].

### 4.5. Enzymatic Assays

To test the peptidase activity of the mutant *Pba* Svx Pep protein and compare it with peptidase activity of the wild-type protein (*Pba* Svx WT), azocasein was used as the substrate (Sigma-Aldrich, St. Louis, MO, USA). Ten µg of the recombinant proteins (in 100 µL) was incubated (60 min) in 250 µL of 0.5% azocasein in 100 mM Tris-HCl buffer with 1 mM ZnSO_4_ and pH range of 7.0–9.0 and a temperature range of 25–50 °C. The reactions were stopped by the addition of 100 µL of 10% trichloroacetic acid (Sigma-Aldrich, St. Louis, MO, USA). The sediment was removed by centrifugation (5000× *g*, 10 min, 25 °C). Supernatants (400 µL) were mixed with 1.0 N NaOH (133 µL), and the absorbance at 440 nm was measured using the microplate reader CLARIOstar (BMG Labtech GmbH, Ortenberg, Germany). One unit of peptidase activity was defined as the amount of enzyme required to produce an absorbance change of 0.1 per min per 1 mg or per 1 nmol of the corresponding protein. Activities were analyzed in six replicates.

The ability of *Pba* Svx WT to degrade O-glycosylated carrot extensins (obtained as described above), non-glycosylated lectin concanavalin A from *Canavalia ensiformis* (PanEco Ltd., Gorki-Leninskiye uts, Russia), and O-glycosylated porcine gastric mucin type II (Sigma-Aldrich, St. Louis, MO, USA) was tested as follows. Thirty µg of each of the three protein substrates diluted in 100 mM Tris-HCl (pH 7.5) was mixed with 75 pmol (2 µg) of *Pba* Svx WT. The reaction mixtures were supplemented with 1 mM ZnSO_4_ and incubated at 40 °C for 30 min (optimal conditions for *Pba* Svx peptidase activity). Two samples from each reaction mixture were collected: one—immediately after addition of *Pba* Svx WT and the second one—after 30 min incubation. After sample collecting, reactions were stopped by the addition of half the volume of SDS-PAGE sample buffer (0.2 M Tris-HCl, 50% Glycerol, 5% SDS, 0.5 mM DTT, 0.05% bromphenol blue, 5 mM EDTA, pH 6.8), followed by heating at 100 °C for 10 min. The samples were visualized by SDS-PAGE followed by the most appropriate staining method for each substrate: Coomassie Brilliant Blue G250 for concanavalin A [37], periodic acid–Schiff (PAS) for porcine gastric mucin [38], and silver nitrate for carrot extensin [39].

The ability of the mutant *Pba* Svx Pep protein (as well as wild-type *Pba* Svx WT protein) to degrade carrot extensins was analyzed by immunolabeling in three replicates. Carrot extensin (30 µg) in 100 mM Tris-HCl with 1 mM ZnSO_4_ was incubated at 40 °C for 30 min alone, or in the presence of 75 pmol of either *Pba* Svx Pep or *Pba* Svx WT. Samples were applied to an Amersham^TM^ Protran^®^ nitrocellulose membranes (Cytiva, Marlborough, MA, USA) at a volume of 3 μL per spot, after which the membranes were completely air-dried for 30 min to fix the protein mixture. To block non-specific binding sites, the membranes were incubated in a solution of 1% BSA in Tris-HCl buffered saline with Tween-20 (TBST: 20 mM Tris-HCl, 150 mM NaCl, 0.1% Tween-20, pH 7.4) for 60 min at room temperature on a shaker (180 rpm). The membranes were incubated for 1 h at room temperature with primary monoclonal rat antibodies JIM11, JIM20, and LM1 (diluted 1:50 in blocking buffer) [40,41], kindly provided by Prof. Paul Knox (University of Leeds, UK). Membranes were then washed three times with TBST for 5 min each. Incubation with 1:10,000-diluted secondary antibodies (goat anti-rat antibodies conjugated with horseradish peroxidase (Wuhan Servicebio Technology Co., Ltd., Wuhan, Hubei Province, China)) was carried out for 1 h at room temperature, followed by washing the membranes with TBST three times for 5 min each to remove unbound conjugate. For detection, the chemiluminescent substrate was prepared immediately before use by mixing equal volumes of ECL A and ECL B reagents (Wuhan Servicebio Technology Co., Ltd., Wuhan, Hubei Province, China) in a 1:1 ratio. The working solution was evenly applied to the membrane, covering its entire surface, and incubated for 1 min. Excess substrate was removed with filter paper, the membrane was placed between layers of transparent polyethylene film, and the signal was recorded using ChemiDoc Imaging System (Bio-Rad, Hercules, CA, USA), individually selecting the exposure time depending on the luminescence intensity.

### 4.6. Gene Expression Analysis Using qRT-PCR

Tobacco plants (*Nicotiana tabacum* Petit Havana SR1) were grown in soil (Borresources, Kerzhenets, Russia) in 50 mL pots for five weeks at 22 °C under a 16/8 light/dark regime. To assess the ability of the target proteins (*Pba* Svx WT and *Pba* Svx Pep) to induce susceptible ethylene-mediated responses (specifically to induce the expression of ethylene-regulated genes) in plants, fully expanded leaves were infiltrated with the following solutions: (1) 10 mM Tris-HCl with 50 mM NaCl buffer, pH 7.5 (control); (2) 1 µM *Pba* Svx protein in the same buffer; (3) 1 µM mutant form *Pba* Svx Pep in the same buffer. Twelve hours after infiltration, total RNA was extracted from the infiltrated parts of the leaves. Plant material was ground in liquid nitrogen in mortars. The obtained powder was resuspended in 1 mL of ExtractRNA Reagent (Evrogen, Moscow, Russia), and the subsequent procedures were performed according to the manufacturer’s instructions. To eliminate residual genomic DNA, RNA samples were treated with DNase I using a DNA-free kit (Thermo Fisher Scientific, Waltham, MA, USA). RNA quantity and quality were analyzed using a NanoPhotometer NP80 (Implen, München, Germany) and electrophoresis in a 1% agarose gel, respectively. One microgram of total RNA was used for cDNA synthesis using RevertAid reverse transcriptase (Thermo Fisher Scientific, Waltham, MA, USA) according to the manufacturer’s instructions. Two µL of fivefold-diluted cDNA was added to the qRT-PCR mixture. qRT-PCR was performed using the EVA-Green-containing master mix (Syntol, Moscow, Russia) according to the manufacturer’s instructions.

Primers for target and reference genes (Supplementary Table S1) were designed using Vector NTI Version 9 software (Invitrogen, Waltham, MA, USA) and synthesized by Evrogen (Moscow, Russia). PCR was performed under the following conditions: 95 °C for 2 min, followed by 45 cycles at 94 °C for 10 s, 60 °C for 15 s and 72 °C for 30 s. After that, melt curve analysis was performed in the temperature range of 60 to 95 °C. The reactions were run, and changes in fluorescence emission were detected using a CFX96 quantitative PCR system (Bio-Rad, Hercules, CA, USA). The amount of fluorescence was plotted as a function of the PCR cycle using CFX Manager Software vesion 3.1 (Bio-Rad, Hercules, CA, USA). The amplification efficiency (E) for all primers was determined using a dilution series of a pool of cDNAs. Additional controls included the omission of reverse transcriptase to measure the extent of residual genomic DNA contamination and the omission of a template. The targets were genes encoding the ethylene-regulated transcription factor ERF1a (LOC107766165) and 1-aminocyclopropane oxidase (ACO1) (LOC107781126). Plant genes encoding elongation factor 1-α and the β-subunit of ATP synthase were used for normalization of the expression of the target genes. Relative expression levels were determined as the ratios between the quantities of cDNA corresponding to the target and reference genes. Experiments were performed in at least twenty biological replicates. The statistical significance of differences was assessed using a Kruskal–Wallis test followed by Dunn’s post-test, with *p*-value < 0.05 considered statistically significant.

## Data Availability

Data are contained within the article and Supplementary Materials.

## Supplementary Materials

The following supporting information can be downloaded at: https://www.mdpi.com/article/10.3390/ijms27094092/s1.
