# Supplementary material for: What Is the Functional Role of the Acyltransferase-like Domain in the Svx Peptidase of the Phytopathogenic Bacterium Pectobacterium atrosepticum?
Source: Int J Mol Sci. 2026 May 2;27(9):4092. doi: 10.3390/ijms27094092 (PMC13164431; doi:10.3390/ijms27094092)
Supplement: Supplementary file 1 [file ijms-27-04092-s001.zip › ijms-4270474-supplementary.pdf]

Supplementary Materials

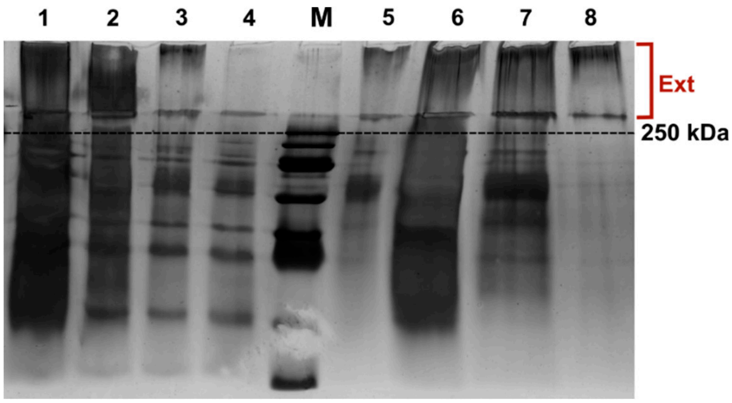

Figure S1. Electroforegram showing protein fractions from plant cell walls of the carrot roots, obtained during cation exchange chromatography performed using CM-sepharose Fast Flow. 1—$CaCl_2$-extracted proteins from carrot plant cell walls; 2—LiCl-extracted proteins from carrot plant cell walls; 3 and 4—proteins, that did not attach to CM-sepharose Fast Flow resin; 5–8—protein fractions eluted from CM-sepharose Fast Flow resin. Ext—extensins. M—molecular weight marker. Gel was visualized by silver staining.

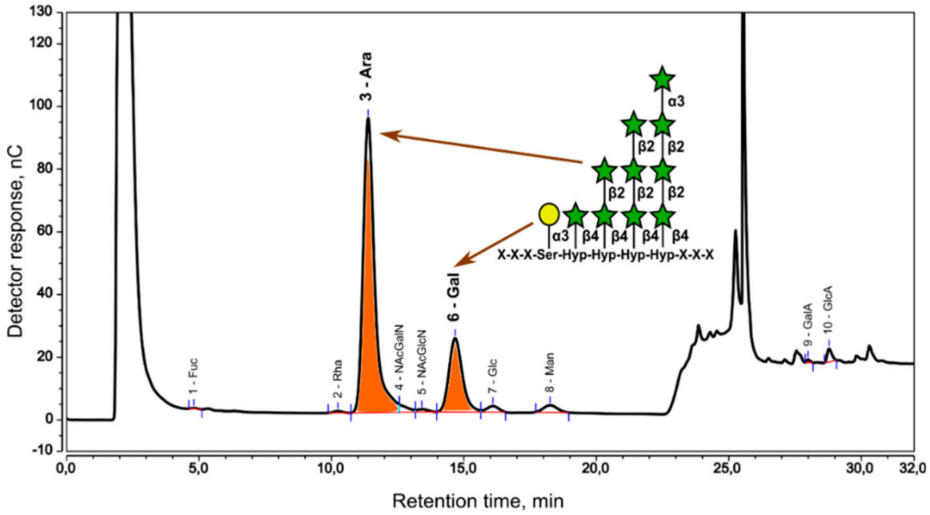

Figure S2. Chromatogram of carbohydrates from purified extensin-rich fraction obtained from carrot roots. Ara—arabinose, Gal—galactose, Fuc—fucose, Rha—rhamnose, NAcGlcN—N-acetylglucosamine, NAcGalN—N-acetylgalactosamine, Glc—glucose, Man—mannose, GalA—galacturonic acid, GlcA—glucuronic acid. The figure also shows the scheme of glycosylation of extensins: the yellow circle is a galactose residue attached to the serine residue; the green stars are arabinans attached to hydroxyproline residues.

Table S1. Primers used for qRT-PCR in the present study.

Name	Primer sequence	Gene ID
NtEF_F	GCCCAACACTTCTTGATGCTC	LOC107791623
NtEF_R	GACACCAGTTTCCACACGACC	
NtATP_F	GGTCGATGGCTTGGGAGTACC	LOC107762397
NtATP_R	GCACAGATTTACGTTCAATAATACCAG	
NtERF1a_F	GATAGGGCAGCTTATTCAATGAGAGGTC	LOC107766165
NtERF1a_R	CCAAACATTCAAATTCAAGAACTTCCC	
NtACO1_F	CTTGAAGCTGTGCAAGCTGAGGTTAC	LOC107781126
NtACO1_R	CCAAGATTTTCACATAGCAAGTCCAG	
